# Whole genome scan reveals the genetic signature of African Ankole cattle breed and potential for higher quality beef

**DOI:** 10.1186/s12863-016-0467-1

**Published:** 2017-02-09

**Authors:** Mengistie Taye, Jaemin Kim, Sook Hee Yoon, Wonseok Lee, Olivier Hanotte, Tadelle Dessie, Stephen Kemp, Okeyo Ally Mwai, Kelsey Caetano-Anolles, Seoae Cho, Sung Jong Oh, Hak-Kyo Lee, Heebal Kim

**Affiliations:** 1 Department of Agricultural Biotechnology, Animal Biotechnology and Research Institute for Agriculture and Life Sciences, Seoul National University, Seoul, 151-921 Korea; 2 Bahir Dar University, College of Agriculture and Environmental Sciences, PO Box 79, Bahir Dar, Ethiopia; 3 National Human Genome Research Institute, National Institutes of Health, 50 South Drive, Building 50 Room 5351, Bethesda, MD 20892 USA; 4 The University of Nottingham, School of Life Sciences, Nottingham, NG7 2RD UK; 5 International Livestock Research Institute (ILRI), PO Box 5689, Addis Ababa, Ethiopia; 6 International Livestock Research Institute (ILRI), PO Box 30709-00100, Nairobi, Kenya; 7 The Centre for Tropical Livestock Genetics and Health, The Roslin Institute, The University of Edinburgh, Easter Bush Campus, Midlothian, EH25 9RG Scotland; 8 Department of Animal Sciences, University of Illinois, Urbana, IL 61801 USA; 9 C&K genomics, Main Bldg. #514, SNU Research Park, Seoul, 151-919 Republic of Korea; 10 National Institute of Animal Science, RDA, Wanju, Republic of Korea; 11 The Animal Molecular Genetics & Breeding Center, Department of Animal Biotechnology, Chonbuk National University, Jeonju, 561-756 Korea; 12 Institute for Biomedical Sciences, Shinshu University, Nagano, Japan

**Keywords:** African cattle, Ankole cattle, Meat quality, Sanga cattle, XP-CLR, XP-EHH

## Abstract

**Background:**

Africa is home to numerous cattle breeds whose diversity has been shaped by subtle combinations of human and natural selection. African Sanga cattle are an intermediate type of cattle resulting from interbreeding between *Bos taurus* and *Bos indicus* subspecies*.* Recently, research has asserted the potential of Sanga breeds for commercial beef production with better meat quality as compared to *Bos indicus* breeds. Here, we identified meat quality related gene regions that are positively selected in Ankole (Sanga) cattle breeds as compared to *indicus* (Boran, Ogaden, and Kenana) breeds using cross-population (XP-EHH and XP-CLR) statistical methods.

**Results:**

We identified 238 (XP-EHH) and 213 (XP-CLR) positively selected genes, of which 97 were detected from both statistics. Among the genes obtained, we primarily reported those involved in different biological process and pathways associated with meat quality traits. Genes (*CAPZB*, *COL9A2*, *PDGFRA*, *MAP3K5*, Z*NF41*0, and *PKM2)* involved in muscle structure and metabolism affect meat tenderness. Genes (*PLA2G2A*, *PARK2*, *ZNF410*, *MAP2K3*, *PLCD3*, *PLCD1*, and *ROCK1*) related to intramuscular fat (IMF) are involved in adipose metabolism and adipogenesis. *MB* and *SLC48A1* affect meat color. In addition, we identified genes (*TIMP2*, *PKM2*, *PRKG1*, *MAP3K5*, and *ATP8A1*) related to feeding efficiency. Among the enriched Gene Ontology Biological Process (GO BP) terms, actin cytoskeleton organization, actin filament-based process, and protein ubiquitination are associated with meat tenderness whereas cellular component organization, negative regulation of actin filament depolymerization and negative regulation of protein complex disassembly are involved in adipocyte regulation. The MAPK pathway is responsible for cell proliferation and plays an important role in hyperplastic growth, which has a positive effect on meat tenderness.

**Conclusion:**

Results revealed several candidate genes positively selected in Ankole cattle in relation to meat quality characteristics. The genes identified are involved in muscle structure and metabolism, and adipose metabolism and adipogenesis. These genes help in the understanding of the biological mechanisms controlling beef quality characteristics in African Ankole cattle. These results provide a basis for further research on the genomic characteristics of Ankole and other Sanga cattle breeds for quality beef.

**Electronic supplementary material:**

The online version of this article (doi:10.1186/s12863-016-0467-1) contains supplementary material, which is available to authorized users.

## Background

Africa, with its diverse agro-ecological zones, is a home to diverse cattle breeds adapted to their local environments. African cattle breeds are derived from *Bos taurus* and *Bos indicus* subspecies introduced to the continent at different times, and through interbreeding between them [1, 2]. Since the introduction, their diversity has been shaped by subtle combinations of human and natural selection. Selection in African cattle is mainly for sociocultural concerns and to survive the heterogeneous environment [3]. African cattle have been evolved to adapt to the poor feed availability, high environmental temperature, and high prevalence of internal and external parasite and disease conditions of the continent. These cattle breeds display better heat tolerance, adaptability, tick resistance, reproductive longevity, and maternal characteristics such as fertility, low inter-calf periods and cow efficiency [1, 4–7].

African Sanga cattle, sometimes referred to as *Bos africanus*, are an intermediate type of cattle believed to be the result of interbreeding between *Bos taurus* and *Bos indicus,* which dwell in eastern, central and southern Africa [1, 8]. Generally, Sanga cattle can be identified by their long and slender horns, small cervicothoracic hump, and small and unfolded dewlap [9]. There are 30 Sanga cattle breeds/strains in Africa subdivided into Sanga of eastern and Sanga of southern Africa based on geographical location [8]. Recently, research outputs are asserting the potential of African Sanga and Sanga derived breeds to produce carcass and meat quality attributes that favorably compare to British and Continental breeds and are often better than those of the *Bos indicus* breeds [6, 10–13]. Sanga breeds in south Africa (eg., Bonsmara, Drakensberger and Nguni) were found to produce beef with lower shear force, shorter myofibrillar fragment length, larger rib fat thickness, and larger soluble collagen when compared with *indicus* (Brahman) cattle [11].

Meat quality is a general term used to describe the attributes of meat which include carcass composition and conformation, the eating quality of meat, health issues associated with meat, and production and environmental issues [14]. Meat sensory characteristics such as tenderness, flavor, juiciness, and color are important meat quality parameters which are affected by biological characteristics and proteolytic activities of muscle [15, 16]. The biological characteristics of muscles such as fiber type, collagen, intramuscular adipose tissue and protease activities regulate meat tenderness and flavor and are known to be affected by genetic and rearing factors [15]. The heritability of beef quality traits is low to moderate which varies between breed groups, methods of estimation, number of records, and other factors [17, 18]. The genetic variation within and between breeds is because of the positive selection of gene regions caused by beneficial polymorphisms in the genes affecting the traits. Identification of selection signatures in the genome provide information about the evolutionary processes involved in shaping genomes and functional information about genes/genomic regions [19].

Studies attempting to detect positive selection signatures in African cattle have reported several genes involved in immune system, reproduction, energy metabolism, coat coloration, thermoregulation and tick resistance [20, 21]. The detection of immune related genes might be related to the selective pressure that has been exerted by the long-term presence of pathogens in the continent [21], whereas signatures of selection associated with reproduction and thermoregulation is an adaptation to perform under heat stress conditions [20]. However, there have been no previous studies attempting to identify genes affecting meat quality traits in African cattle in general and Sanga cattle in particular.

In this study, we reported genes that are positively selected in Ankole cattle population associated with meat quality traits. This was done by scanning the whole genome of four African cattle breeds (African Sanga cattle: Ankole; and three *indicus* breeds: Boran, Ogaden, and Kenana) [22, 23]. We employed cross-population extended haplotype homozygosity (XP-EHH) and cross population composite likelihood ratio (XP-CLR) statistics in order to detect selection signatures from different data patterns; two approaches were used as each has its own advantages. XP-EHH compares haplotype lengths of populations to detect selective sweeps when the allele has approached or achieved fixation in one population but remains polymorphic in the other population [24]. XP-CLR is a statistic based on allele frequency differentiation across populations. It is not affected by ascertainment biases and has the advantage of being able to detect older signals and selection on standing variation [25].

## Results and discussion

### Data description

DNA samples extracted from whole blood samples of four African cattle breeds (Boran, Ogaden, Kenana and Ankole) were sequenced to ~ 11 × genome coverage each. Using a standard sample preparation and whole genome re-sequencing pipeline, an overall alignment rate of 98.84% covering 98.56% of the taurine reference genome was obtained. After filtering false positive calls using several filtering steps, a total of ~37 million SNPs were retained and used for detection of positive selection signature analysis.

### Phylogenetic tree

**Fig. 1:**
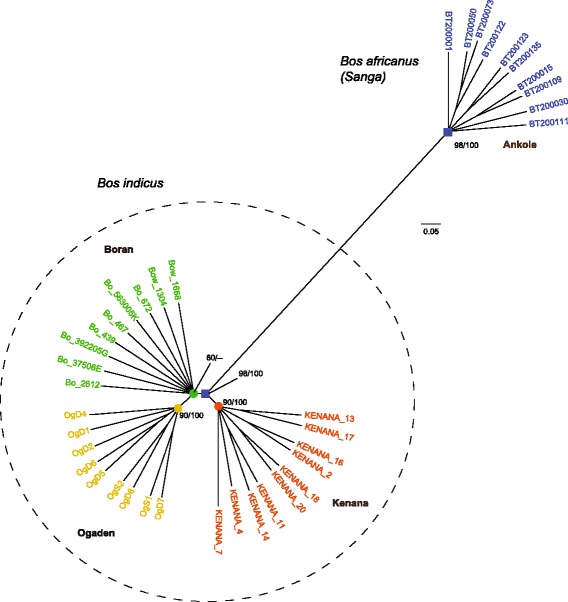
Maximum likelihood phylogenomic tree derived from autosomal SNPs of 38 African cattle individuals. The data set (26,427,196 base pairs) was analyzed with maximum likelihood (ML) and neighbor-joining (NJ) methods which revealed identical topologies. The robustness of the phylogenomic analysis is indicated to the respective nodes: left numbers are bootstrap values for ML tree and right ones are quartet puzzling reliability values for NJ tree

Maximum likelihood (ML) and neighbor-joining (NJ) methods produced consistent features regarding the genetic distance between the breeds considered (Fig. 1). Ankole cattle are clearly separated from the three *indicus* breeds (100% bootstrap values/quartet puzzling reliability values). Within *indicus* cattle, each of the three breeds were also depicted as a monophyletic group with highly significant values.

While African *indicus* cattle have been present on the continent since 1500 BC [8], Sanga cattle were derived through hybridization between taurine cattle and zebu cattle around 700 AD [1]. More than 30 breeds/strains of Sanga cattle can be found distributed throughout Eritrea, Ethiopia, southern Sudan, the Great Lakes region of East Africa and southern Africa. The Ankole group is one of the three groups of Sanga cattle representing Sanga cattle in Uganda, Rwanda, Burundi, Tanzania and Democratic Republic of Congo [8]; it is a valuable and widely used genetic resource in these regions.

### Positive selective signature in Ankole cattle population

XP-EHH and XP-CLR tests were performed in order to detect positive selection signatures in Sanga (Ankole) cattle. The genome of Ankole population was compared with the genomes of three *indicus* cattle breeds grouped together into one population. Based on the analysis, we obtained 238 and 213 putatively advantageous positively selected genes from XP-EHH (Additional file 1: Table S1) and XP-CLR test statistics (Additional file 2: Table S2), respectively; of these, 98 genes were detected in both statistics (Additional file 3: Table S3). Gene Ontology Biological Processes (GO BP) and Kyoto Encyclopedia of Genes and Genomes (KEGG) pathways within DAVID were used to build on biological modules consisting of clusters of functional terms [26]. All the 354 genes obtained from both XP-EHH and XP-CLR statistics were included, after removing duplicates, for the analysis. Gene ontology analysis resulted in 44 significantly (*p* < 0.05) enriched GO BP categories (Additional file 4: Table S4) and the KEGG-pathway analysis resulted in three significantly enriched pathways (*p* < 0.05; Table 1). The ClueGO plugin [27] created a functionally organized pathway term networks (Fig. 3).

**Table 1:** KEGG pathways obtained from DAVID gene enrichment (*p* < 0.05) analysis

KEGG pathway term	*P*-value	Genes	Fold enrichment
Leukocyte trans-endothelial migration	0.019	*ROCK1, PIK3CB, CLDN10, TXK, RAPGEF4, RAPGEF3, CTNNA3*	3.24
MAPK signaling pathway	0.021	*MAP4K4, ACVR1B, FGF18, MAP3K5, MAP2K3, MRAS, PDGFRA, PLA2G2A, NR4A1, MAPKAPK2, NFATC2*	2.25
Melanoma	0.038	*FGF18, PIK3CB, MITF, PDGFRA, MDM2*	3.85

We used 354 genes obtained from both XP-EHH and XP-CLR statistics after removing duplicate genes

We also analyzed Tajima’s D for the candidate gene regions which revealed a significant departure from neutrality and indicated the selective maintenance of alleles within the Ankole population as compared to its *indicus* counterparts (Table 2). The negative Tajima’s D values obtained for the candidate gene regions indicate the presence of an excess of rare alleles in the population. It is known that low-frequency alleles contribute less to the number of pair-wise differences in a sample set than alleles of moderate frequency do; a surplus of rare alleles inflates the latter value disproportionately to the former value [28]. Similarly, population differentiation analysis supported the positive selection of candidate genes (Table 2); candidate gene regions produced higher values of fixation index [29]. F_ST_ has been widely used to identify selective sweep regions in different livestock species [30]. The Tajima’s D and F_ST_ plot of candidate gene regions are presented in Fig. 4 and Additional file 5: Figure S1.

**Table 2:** Summary of major candidate genes related to meat quality characteristics and feed intake in Sanga cattle population detected by XP-EHH and XP-CLR statistics (see Additional file 1: Table S1 and Additional file 2: Table S2)

Candidate genes	Chr.	Window (Mbp)	XP-CLR	Max XP-EHH	XP-EHH *P*-value	Tajima’s D	Weighted F_ST_	Species, trait and reference
*PIK3CB*	1	131.50–131.55	121.48	2.21	1.69.E-03	−1.06	0.27	RFI [79]^a^
*MRAS*	1	131.73–131.78	112.25	-	-	−1.87	0.16	FCE [79]^a^
*PLA2G2A*	2	133.28–133.33	117.48	-	-	0.70	0.27	IMF [58]^b^, [59]^c^
*CAPZB*	2	133.78–133.83	142.50	-	-	−0.47	0.22	Tenderness [32]^c^
*COL9A2*	3	106.38–106.43	107.14	1.93	5.95.E-03	−1.30	0.15	Tenderness [46]^b^
*LIMA*	5	29.78–29.83	117.18	-	-	−0.33	0.39	Tenderness [45]^b^
*SLC48A1*	5	32.63–32.68	101.45	1.80	6.96.E-03	−0.35	0.26	Meat color [68]^d^
*MB*	5	74.18–74.23	129.86	1.96	5.00.E-03	−0.98	0.20	Meat color [41, 67]^d^
*APOL6*	5	74.20–74.25	-	2.00	5.00.E-03	−0.91	0.13	IMF [65]^d^
*ATP8A1*	6	62.90–62.95	264.27	2.02	4.00.E-03	−1.39	0.36	FCE [76]^c^
*PDGFRA*	6	71.35–71.40	408.60	2.61	3.00.E-04	−2.29	0.45	Tenderness [53]^b^
*MAP3K5*	9	75.55–75.60	147.37	2.09	3.00.E-03	−0.23	0.15	RFI [75]^b^
*PARK2*	9	99.13–99.18	132.40	2.34	2.36.E-03	−0.27	0.30	IMF [62]^a^
*PKM2*	10	18.95–19.00	-	1.97	2.00.E-03	−1.18	0.25	Tenderness [32]^d^, [52]^a^; IMF [64]^b^; Drip loss [70, 71]^b^
*ZNF410*	10	85.68–85.73	165.86	2.18	1.94.E-03	−0.67	0.28	Tenderness [32]^c^, [35]^b^
*AHSA1*	10	89.70–89.75	-	2.23	2.00.E-03	−1.47	0.31	IMF [66]^c^
*MAP4K4*	11	6.65–6.70	-	1.85	9.00.E-03	0.72	0.08	Drip loss [70, 71]^b^
*NFATC2*	13	80.00–80.05	-	2.01	4.24.E-03	2.53	0.25	Tenderness [55]^d^
*WWP1*	14	78.63–78.68	133.90	-	-	1.03	0.18	Tenderness [50]^d^
*OR2D2, OR10A4, OR2D3*	15	46.40–46.45	-	2.07	3.45.E-03	2.61	0.18	Feed intake [78]^d^; [77]^b^
*MAP2K3*	19	35.85–35.90	-	1.93	4.00.E-03	−0.20	0.16	IMF [49]^b^
*PLCD3*	19	45.40–45.45	-	1.89	5.00.E-03	−0.18	0.03	IMF [56]^d^
*TIMP2*	19	54.10–54.15	-	1.88	7.46.E-03	−0.73	0.18	RFI [73]^c^
*PLCD1*	22	11.45–11.50	195.30	2.12	3.00.E-03	−1.43	0.14	IMF [56]^d^
*ROCK1*	24	35.48–35.53	130.40	2.22	1.61.E-03	0.93	0.29	Tenderness [48]^a^; IMF [61]^b^
*PRKG1*	26	7.43–7.48	166.61	-	-	−1.27	0.13	Tenderness [54]^d^; IMF [36]^b^; RFI [72]^c^

Note: *Chr.* Chromosome, Window: start and end positions of the gene region; *RFI* residual feed intake, *FCE* feed conversion efficiency, *IMF* intramuscular fat; Superscripts in the Species, Trait and Reference column indicate the species that the trait has been previously reported for as ^a^chicken, ^b^pork, ^c^beef, ^d^general (not for a specific species)

### Biological process and pathways related to meat quality traits

Meat quality is a multifactorial and complex trait affected by different factors at different levels ranging from molecular to mechanical. Molecularly, genes involved in many cellular mechanisms such as muscle growth, glycolysis, muscle contraction, stress reaction, cell cycle, proteolysis, protein ubiquitination and apoptosis have been reported to be associated with meat quality characteristics [16, 31, 32]. Previous studies reported that, as compared with *indicus* breeds, Sanga breeds produce better quality beef [11–13] with lower shear force, shorter myofibrillar fragment length, larger rib fat thickness, larger soluble collagen, and higher percent drip loss [11]. Additionally, Sanga cattle have better feed conversion efficiency, reproductive performances, and tick resistance in the tropics [33].

**Fig. 2:**
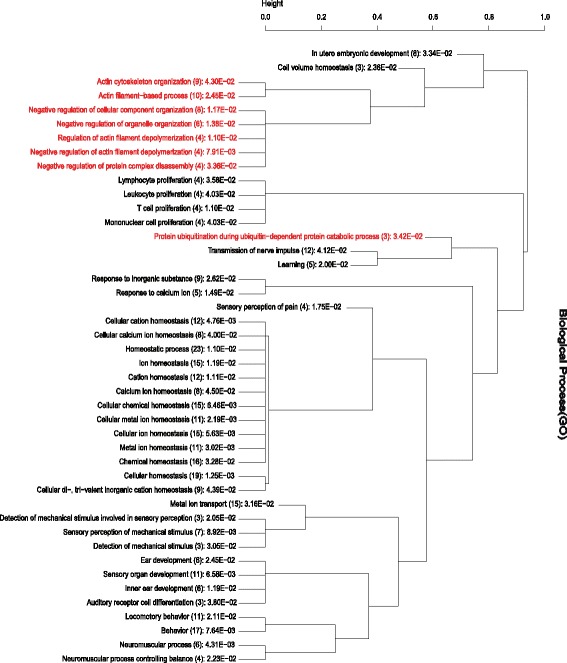
Functional clustering of GO BP terms annotated from DAVID gene ontology analysis. All the 44 significantly (*p* < 0.05) enriched BP terms were used for the functional clustering. Clusters related to meat quality characteristics are highlighted in *red color*

From DAVID gene ontology analysis, 44 significant (*p* < 0.05) GO BP terms were enriched (Fig. 2; Additional file 4: Table S4). The BP terms and gene clusters related to meat quality characteristics were chosen based on their biological function and previous literature. Accordingly, among the enriched GO BP terms (Fig. 2), actin cytoskeleton organization (represented by nine genes; *FMNL1, FMNL3, DOCK2, LIMA1, ROCK1, MRAS, PRKG1, CAPZB,* and *ADD1*) and actin filament-based process (additionally contains *MYO7A*) are related to meat tenderness [32, 34, 35]. Cellular component organization, a cellular level process which results in the assembly and arrangement of constituent parts or disassembly of a cellular component, is important for beef tenderness [32]. It is also significantly differentially expressed in relation to pork IMF and tenderness [36]. Five genes (*WWP1*, *MDM2*, *CAND1*, *PARK2,* and *LNX1*) were involved in protein ubiquitination, which is a key step in protein degradation [37]. Ubiquitination pathway affects muscle properties that are relevant for the quality of meat at postmortem [38], and are expressed in relation to tenderness [36]. GO terms of negative regulation of actin filament depolymerization and negative regulation of protein complex disassembly are involved in adipocyte regulation [34].

**Fig. 3:**
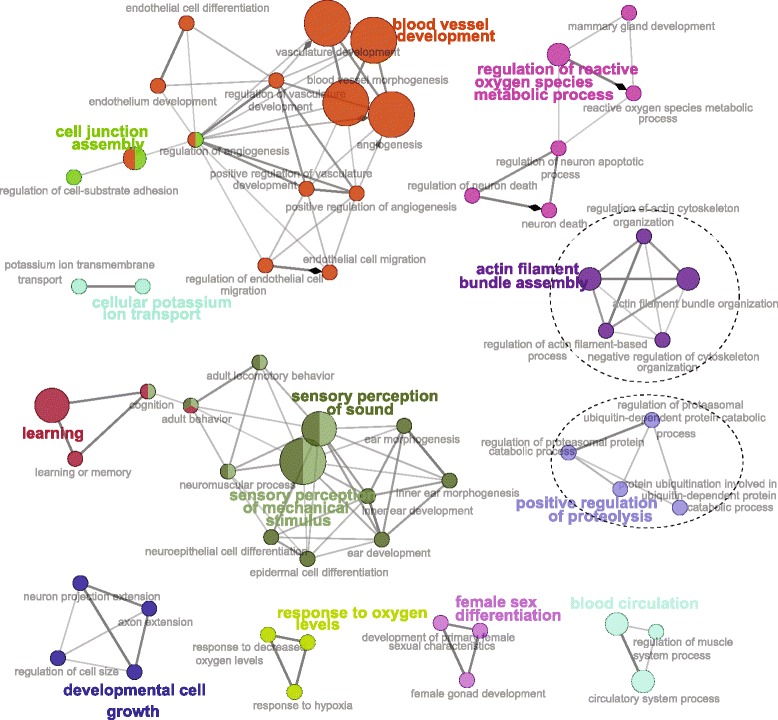
ClueGO gene ontology analysis of 354 positively selected genes in Ankole cattle population. ClueGO visualizes the selected terms in a functionally grouped annotation network that reflects the relationships between the terms based on the similarity of their associated genes. Nodes represent gene ontology terms to which their size reflects the statistical significance of the terms. The most prominent gene ontology term for each group is highlighted in *colors*, and the *circled* gene ontology terms are related to meat quality characteristics

The KEGG MAPK pathway (*p* = 0.0215; Table 1), represented by eight genes (*MAP4K4*, *ACVR1B*, *FGF18*, *MAP3K5*, *MAP2K3*, *MRAS*, *PDGFRA*, *PLA2G2A*, *NR4A1*, *MAPKAPK2*, and *NFATC2*) is responsible for cell proliferation and plays an important role in hyperplastic growth [39], which has a positive effect on meat tenderness [31]. Gap junction, regulation of actin cytoskeleton and MAPK signaling pathway also are important in residual feed intake [40]. The ClueGO plugin created a functionally organized pathway term network (Fig. 3), that the networks actin filament bundle assembly and positive regulation of proteolysis were among enriched networks in relation to meat quality characteristics [32].

### Genes affecting meat quality traits in Ankole cattle

Here, we described genes positively selected in Ankole Sanga cattle that are potentially associated with meat quality and feed conversion efficiency traits based on previous studies and their biological functions (Table 2; Additional file 6: Table S5).

#### Genes related to meat tenderness

Meat tenderness is an important meat eating quality trait. It is mainly affected by the quantity and solubility of connective tissue, composition and contractile state of muscle fibers, and the extent of proteolysis in rigor muscle [11, 31, 41]. Tender meat contains higher levels of soluble collagen, more fat, and lower water content. Myofibril fragmentation index also has a positive correlation with beef loin tenderness [42]. Sanga breeds have a lower percentage of white muscle fiber and a higher myofibrillar fragmentation index [6, 11], which results in lower shear force and more tender beef compared to *indicus* cattle [11]. In this study, we have identified genes (*CAPZB, COL9A2, PDGFRA, MAP3K5, ZNF410, LIMA1,* and *PKM2*) that may potentially affect muscle structure and development thereby affecting meat tenderness in Ankole cattle.

**Fig. 4:**
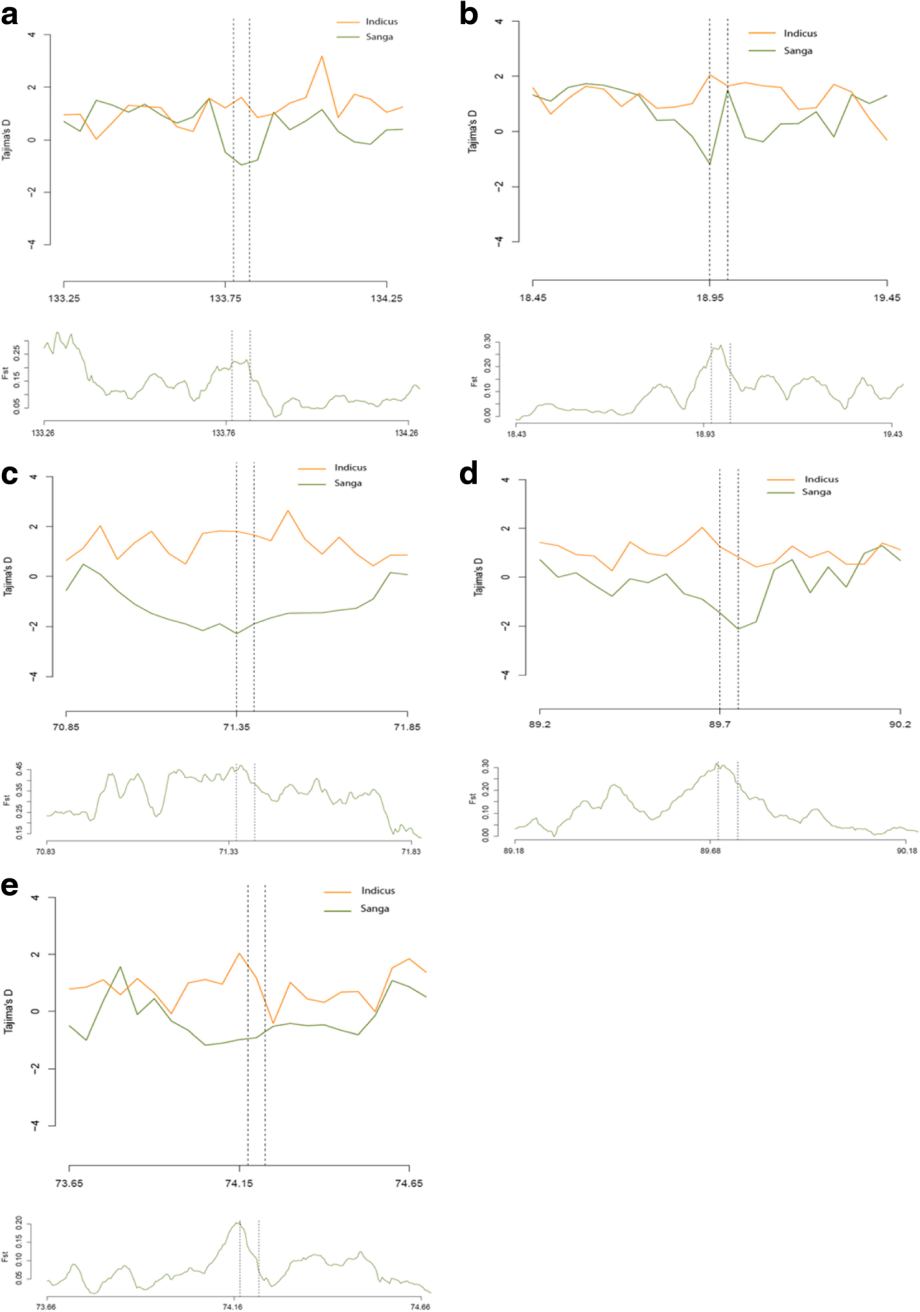
Tajima’s D and F_ST_ plot of positively selected gene regions in Sanga and *indicus* cattle populations. **a** *CAPZB* gene; **b** *PKM2* gene; **c** *PDGFRA* gene*;* **d** *AHSA1* gene; and **e** *MB* gene. For other genes, please see Additional file 6: Figure S1. The Tajima’s D plot for each gene region (upper plot for each gene) show the Tajima’s D value within a 50 kb window plotted for both populations. The smaller (negative) Tajima’s D value in the Sanga population shows that the gene region considered is under positive selection. The F_ST_ plot (lower plot for each gene) represents the F_ST_ values calculated within 50 kb windows separated by 5 kb window steps

The *CAPZB* (XP-CLR = 142.50) gene encodes the beta subunit of the barbed-end actin binding protein, which belongs to the F-actin capping protein family. It is involved in skeletal muscle development and growth [43], and cell signaling and regulation of actin in myofilament contractility [38]. When up-regulated, it increases the ability of muscle accretion in pigs [43]. *CAPZB* contributes to muscle metabolic and structural properties and proteolytic processes providing a link between these functional networks which are important for maturation of muscle to meat [38]. A previous functional analysis of meat tenderness revealed a positive correlation between *CAPZB* expression and beef tenderness [32]. In the pig, *CAPZB* is an essential element for protein kinase signaling to the myofilaments and, as a structural protein, it has been shown to influence muscle biochemistry and its postmortem abundance is related to meat quality [44]. The Tajima’s D and F_ST_ plot of the *CAPZB* gene region (Fig. 4a) shows the presence of an excess of rare alleles in Ankole population and the differentiation of the region between the compared breeds, respectively.

*LIMA1* (called *EPLIN)* encodes a cytoskeleton-associated protein that inhibits actin filament depolymerization and cross-links filaments in bundles. It is associated in pigs with functions regarding muscle development and metabolism [45]. *ZNF410*, also known as *APA-1*, is an essential component of the stress pathway involved in the meat tenderization process [32]. In previous muscle transcriptome analyses, *ZNF410* has been shown to be highly expressed in the longissimus muscle of Basque pigs that are known to produce pork with higher intramuscular fat and tenderness compared to Large White pigs [35]. *COL9A2,* a fibrillar collagen, constitutes the largest component of extracellular matrix (ECM) to which its amount, type, and solubility present in muscle tissue have a strong effect on meat tenderness [39]. This gene was found to be upregulated in the longissimus dorsi muscle of Jeju native piglets [46], whose meat is known for its preferable taste, tenderness and superior marbling [47]. *ROCK1*, a gene that regulates actin cytoskeleton and cell polarity, is associated with body weight, carcass weight, shank length, shank circumference and other carcass weight traits in chicken [48].

Genes involved in MAPK signaling (*MAP3K5, MAP2K3, MAP4K4,* and *MAPKAPK2*) were also identified. MAPK signaling is one of the major intracellular signaling pathways affecting myogenesis [49] and is relevant to postmortem meat quality [38]. *MAP2K3* shows associations with loin muscle area and fat traits in pigs, implying roles in muscle differentiation and growth [49].

E3 ubiquitin ligase genes (*WWP1,* and *PARK2*) play an important role in the regulation of a wide variety of cellular functions such as protein degradation, transcription, and RNA splicing. These genes catalyze protein ubiquitylation resulting in the targeting of proteins toward various cellular fates, with proteasome-mediated proteolytic degradation [50]. The ubiquitin–proteasome system is one of the proteolytic systems responsible for the majority of the protein degradation in muscle that is relevant for meat quality postmortem [51].

The expression of *PKM2* (XP-EHH = 1.9696; *p* =2.00.E-03), a gene involved in energy metabolism, is positively correlated with WBSF and has been reported as a functional protein marker for meat tenderness in Thai indigenous chicken [52] and beef [32]. *PDGFRA* also has an effect on shear force and Loin Eye Area in pig [53]. The Tajima’s D and F_ST_ plot of *PKM2* and *PDGFRA* gene regions is shown in Fig. 4b and c, respectively. *PRKG1* is reported to be important in the conversion of muscle to meat [54]. *NFATC2* is a calcineurin substrate expressed in skeletal muscle which is responsible for activating new myotubes [39]. Calcineurin is crucial for myocyte differentiation and determination of the slow oxidative fibre phenotype [55].

#### Genes related to meat intramuscular fat (IMF)

IMF is a heritable meat quality trait which affects flavor, juiciness, visual characteristics and meat tenderness. It is positively correlated with body fat and red muscle fiber [41]. Steak from Ankole cattle has been found to be juicy than those Ankole-Boran crossbreds [13], and Strydom et al. showed higher levels of rib fat thickness in Sanga as compared to indicus cattle [10].

We identified several genes (*PLA2G2A*, *PARK2, ZNF410, PKM2, MAP2K3, PLCD3, PLCD1, ROCK1,* and *AHSA1*) which affect the fat content of meat in Ankole cattle. *PLA2G2A* (XP-CLR = 117.48) is a member of the phospholipase A2 family (*PLA2*), which is involved in the hydrolysis of phospholipids into fatty acids and phosphatidylinositol and phospholipid metabolism [56]. Also referred to as Adipose-Specific Phospholipase A2 (*AdPLA*), it is involved in adipocyte metabolism and catalyzes the efficient release of free fatty acids and lysophospholipid from phosphatidylcholine [57]. It has been reported in the literature that *PLA2* has a positive effect on porcine fat deposition (IMF) and potentially regulates lipolysis and increases the MUFA deposition rather than the SFA deposition [58]. It is also associated with intramuscular fat in beef cattle [59]. *Pla2g2a* has been reported to be a candidate gene in relation to obesity in mice [60].

*ROCK1* is involved in pathways relevant to muscle/adipose tissue function in pigs with divergent phenotypes for fatness traits [61]. E3 ubiquitin ligase enzymes have been identified to be involved in the modulation of lipid biology [50, 62]. *PARK2* is a strong positional candidate for adiposity in chicken and a positive regulator of fat metabolism [62]. *PRKG1* is involved in gap junction and is a candidate gene for intramuscular fat in the pigs [36]. *MAP2K3* has been shown to be associated with loin muscle and fat traits in pigs [49]. *MAP4K4* is involved in adipogenesis, triglyceride storage, fatty acid release, fatty acid oxidation and mitochondrial oxidative phosphorylation [63]. *PKM2* is significantly associated with back fat thickness, an economically important trait in pigs [64]. *APOL6* is one of the most important known genes involved in lipoprotein metabolism [65]. Phospholipase C family genes (*PLCD1* and *PLCD3*) generate diacylglycerol and are involved in phosphatidylinositol catabolism and phospholipid synthesis [56]. The transcription of *AHSA1* (*AHA1*) is related to Omega-3 fatty acids in skeletal muscle, which influence meat tenderness, juiciness, and flavor, and are beneficial to human health [66]. The positive selection of the *AHSA1* gene region is shown in the Tajima’s D and F_ST_ plot in Fig. 4d.

#### Genes related to meat color, drip loss, and feed conversion efficiency (FCR)

Meat color and water holding capacity of meat are among the quality parameters used as an indicator of freshness and wholesomeness [41, 67]. These characteristics are related to variations in the glycolysis rate and muscle temperature decline postmortem. Myoglobin (*MB*; XP-CLR = 129.86; XP-EHH = 1.9640; *p* = 5.00.E-03), a globular single chain protein located in the sarcoplasm, is the principle protein responsible for the red color of meat. *MB* serves as a reserve supply of oxygen and facilitates the movement of oxygen within muscles [41, 67]. Figure 4e shows the Tajima’s D and F_ST_ plot of *MB* gene region in Sanga and *B. indicus* populations. The Solute Carrier Family 48 (Heme Transporter), Member 1 (*SLC48A1*) is responsible for the transport of heme from endosome to the cytosol [68] and may also have a function in meat color. In general, beef from Sanga cattle breeds showed higher chroma than that of *indicus* cattle breeds [11].

The loss of reddish fluid mainly consisting of water and proteins from meat, called drip loss, is an important meat quality characteristics which is affected by several ante- and post-mortem factors [69]. A small but significant difference in drip loss is reported between Sanga and *indicus* cattle breeds; meat from Sanga cattle showed higher drip loss [10, 11]. Higher expression of *PKM2* and *MAP4K4* suppresses the glucose content of muscle cells promoting the onset of anaerobic production of lactate post mortem, thereby facilitating the decline in pH resulting in higher drip loss [70, 71].

Feed intake and efficiency, measured as residual feed intake (RFI), are economically important traits affecting the cost of beef production [72]. Variation in RFI (animals with lower RFI are more efficient) has a genetic component with moderate heritability [73]. We identified positively selected genes (*TIMP2*, *PKM2*, *PRKG1*, *MAP3K5*, and *ATP8A1*) that are reported in the literature to be related to RFI and feed conversion efficiency. *TIMP2* has been shown to be upregulated in low RFI animals in gene expression profiling studies on genes expressed differentially in cattle with high and low RFI [73]. *PKM2* was associated with average daily gain, and feed to body weight gain ratio, with a significant additive and/or dominance effects on these traits [74]. *PRKG1* is involved in gap junction and is also a candidate gene for RFI in cattle [72]. *MAP3K5*, also known as apoptosis signal-regulating kinase 1 (*ASK1*), is a candidate gene for residual feed intake in pigs [75]. *ATP8A1* is also related to feed intake, feed conversion ratio, residual feed intake and weight gain [76]. Olfactory receptor genes (*OR2D2, OR10A4,* and *OR2D3*) have been shown to affect the perception of taste and smell [77, 78] and therefore can be related to feed intake and feeding behavior [77]. *PIK3CB*, and *MRAS* genes involved in the Akt/PI3K and MAPK signaling pathways, respectively, are important for high feed efficiency in chicken [79]. The positive selection of these genes may provide clues as to why Ankole cattle are able to use and survive on poor quality feed and withstand severe droughts [80].

### Implication of the results of this study on Ankole population

The Ankole group is one of the three groups of Sanga cattle representing Sanga cattle in east and central Africa [8]. Ankole breed is a valuable and widely used genetic resource in the region due to its better adaptability. However, there have been no well-designed breed improvement programs for Ankole and other Sanga breeds of eastern Africa [80, 81]. Selective breeding efforts in other South African Sanga cattle breeds (e.g., Mashona, Tuli, and Afrikander) have resulted in local cattle showing higher beef productivity [82]. As cattle genetic resources are being depleted [1, 3] and given the importance of this vital genetic resource, designing breeding programs that would help improve and conserve Ankole cattle is crucial [81]. With this regard, the results provide a basis for further research on the genomic characteristics of Ankole cattle in relation to meat quality traits.

### Limitations of the present study

As is typical in this kind of study, there is a possibility of obtaining false positive results. Therefore, validation with other methods such as GWAS, candidate gene approach and gene expression analysis are suggested. In addition, given the multifactorial nature of meat quality traits, limited published literature is available on genes affecting beef quality characteristics.

## Conclusions

Results from the whole genome scan revealed several positively selected genes involved in different biological and cellular functions including those affecting meat quality characteristics. The genes identified in relation to meat quality characteristics are involved in muscle and lipid metabolism that affect tenderness and intramuscular fat content of meat; and help to improve our understanding of the biological mechanisms controlling meat quality traits in beef cattle production. These results provide a basis for further research on the genomic characteristics of Ankole and other Sanga cattle breeds for quality beef production.

## Methods

### Sample preparation and whole genome re-sequencing

The data used for this paper was obtained from a project: “*The genome landscape of indigenous African cattle*” [Kim *et al.,* *accepted*]. DNA extracted from whole blood samples (10 ml) taken from four African cattle breeds (ten Ankole, nine Boran, nine Ogaden and ten Kenana) was used for this analysis. G-DEXTMIIb Genomic DNA Extraction Kit (iNtRoN Biotechnology, Seoul, Korea) was used to isolate DNA according to the manufacturer’s protocol. To generate inserts of ~300 bp, 3 μg of genomic DNA was randomly sheared using Covaris System. Using the TruSeq DNA Sample Prep. Kit (Illumina, San Diego, CA), we constructed the library following the manufacturer’s guidelines and whole genome sequencing was performed using the Illumina HiSeq 2000 platform. To check the quality of the raw sequence data, we used fastQC software [83]. Pair-end sequence reads were mapped to the reference bovine genome (UMD 3.1) using Bowtie2 [84] with default parameters except the “--no-mixed” option. The overall alignment rate of reads to the reference sequence was 98.50% with an average read depth of 10.8×. On average across the whole samples, the reads covered 98.51% of the genome.

We used open source software packages of Picard tools (http://broadinstitute.github.io/picard), SAMtools [85], and Genome Analysis ToolKit 1.4 (GATK) [86] for downstream processing and variant calling. Picard tools was used to filter potential PCR duplicates. SAMtools was used to create index files for reference and bam files. Genome analysis toolkit 1.4 performed local realignment of reads to correct misalignments due to the presence of indels (“RealignerTargetCreator” and “IndelRealigner” arguments). We used the “UnifiedGenotyper” and “SelectVariants” arguments of GATK to call candidate SNPs. To filter variants and avoid possible false positives, the “VariantFiltration” argument of the same software was adopted with the following options: 1) SNPs with a phred-scaled quality score of less than 30 were filtered; 2) SNPs with MQ0 (mapping quality zero; total count across all samples of mapping quality zero reads) > 4 and quality depth (unfiltered depth of non-reference samples; low scores are indicative of false positives and artifacts) < 5 were filtered; and 3) SNPs with FS (Phred-scaled *P*-value using Fisher’s exact test) >  200 were filtered since FS represents variation on either the forward or the reverse strand, which is indicative of false positive calls. BEAGLE [87] was used to infer the haplotype phase and impute missing alleles for the entire set of cattle populations simultaneously. After all the filtering processes, a total of ~37 million SNPs were retained and used for further analysis.

### Phylogenetic reconstruction

To understand the genetic distance between the breeds considered, we conducted phylogenomic analyses using neighbor-joining (NJ) and maximum likelihood (ML) methods. A total of 26,427,196 autosomal SNPs from the genomes of 38 individuals of four breeds were used for the phylogenic tree construction.

ML analyses [88] were performed using the program TREE-PUZZLE 5.2 [89] with the GTR model. For the quartet puzzling method (1000 puzzling steps), nucleotide frequencies and Ts/Tv ratios (3.18) were estimated from the dataset. Quartet puzzling provided reliability values for maximum likelihood analysis [90].

NJ analysis [91] was performed using the PHYLIP package 3.69 [92] based on Kimura’s [93] 2-parameter distance. Ts/Tv ratios (3.18) were estimated from the dataset using TREE-PUZZLE 5.2 [89] and were used as inputs for the SEQBOOT, DNADIST, NEIGHBOUR, and CONSENS programs of the PHYLIP package. A bootstrap test (with 1000 pseudoreplicates) [94] was performed to obtain statistical support for each node of the NJ tree.

### Detection of positive selection signals

To detect genome-wide selective sweeps, we used the XP-EHH [24] and XP-CLR [25] statistics. XP-EHH assesses haplotype differences between two populations and is designed to detect alleles that have increased in frequency to the point of fixation or near fixation in one of the two populations being compared [24, 95].

We compared the genome of Ankole cattle, used as a test population, with indicus cattle (Boran, Ogaden, and Kenana grouped into one population), used as a reference population. XP-EHH compared the integrated EHH between the two populations for each SNP and the sign of the XP-EHH score determines the direction of selection with extreme values indicating selection in the test population genome. To facilitate comparison of genomic regions across populations, we then split the genome into non-overlapping segments of 50 kb and computed the maximum XP-EHH score in each segment. In order to define the empirical *P*-value, genomic windows were binned in increments of 500 SNPs (combining all windows ≥ 1000 SNPs into one) according to the method used previously [95]. Regions with *P*-values less than 0.01 (1%) were considered strong signals in the Ankole population.

We also performed XP-CLR to identify potential regions differentially selected between the two populations [25]. XP-CLR is a likelihood method for detecting selective sweeps that involve jointly modeling the multilocus allele frequency differentiation between two populations. XP-CLR scores were calculated using XP-CLR software package [25]. We used non-overlapping sliding windows of 50 kb, maximum number of SNPs within each window as 600, and correlation level from which the SNPs contribution to XP-CLR result was down weighted to 0.95. The regions with the XP-CLR values in the top 1% of the empirical distribution (XP-CLR > 97.86) were designated as candidate sweeps and the genes that span the window regions were defined as candidate genes [22]. Significant genomic regions identified from XP-EHH and XP-CLR were annotated to the closest genes (UMD 3.1).

In order to confirm the positive selection of detected genes using these two statistics, we calculated Tajima’s D and F_ST_ for the candidate gene regions. Detecting the same gene regions using different methods can provide cogent evidence for selective influences in the region [30]. Tajima’s D is used to detect selective sweeps going to fixation in the population that makes rare alleles in excess in the population, which results in a negative Tajima’s D [28]. Population differentiation (F_ST_) is based on the principle that natural selection can change the amount of differentiation between different populations of a species. When populations are differentiated, the amount of genetic differentiation within the region that includes selected locus will increase during when the genetic differentiation in the genomic region is greater than the level expected under neutrality, which can be a consequence of natural selection [29]. VCFtools was used in a window size of 50 kb at an interval of 5 kb steps to calculate the Tajima’s D and F_ST_ values of the candidate gene regions [96].

### Characterization of candidate genes under selection

We used the Database for Annotation, Visualization, and Integrated Discovery (DAVID; version 6.7) gene ontology and annotation tool for gene enrichment analysis to further understand the biological functions and pathways of selected genes [26]. Significant GO terms provide insight into the functional characteristics of annotated genes. The KEGG database was also cross-referenced within DAVID to identify significant pathways. R software (version 3.2.1) was used for hierarchical clustering of GO terms from DAVID. Additionally, Cytoscape software’s (version 3.2.0) ClueGO plugin was used to visualize the integration of Gene Ontology (GO) terms as well as KEGG pathways and create a functionally organized GO/pathway term network [27] with default settings.

## Additional files

Additional file 1: Table S1. A summary of genes from Sanga vs. Zebu *(B. Indicus)* XP-EHH test statistics comparison. (XLS 64 kb)
Additional file 2: Table S2. A summary of genes from Sanga vs. Zebu *(B. Indicus)* XP-CLR test statistics comparison. (XLS 54 kb)
Additional file 3: Table S3. Summary of genes common for both XP-EHH and XP-CLR test statistics. (XLS 45 kb)
Additional file 4: Table S4. Gene Ontology Biological Process terms obtained from DAVID gene ontology analysis using all XP-EHH and XP-CLR gene lists. (XLS 47 kb)
Additional file 5: Figure S1. Tajima’s D and F_ST_ plot of positively selected gene regions in Sanga and *indicus* cattle populations. The Tajima’s D plot for each gene region (upper plot for each gene) is the Tajima’s D value within 50 kb window plotted for both populations. The smaller (negative) Tajima’s D value in Sanga population shows that the gene region considered is under positive selection. The F_ST_ plot (lower plot for each gene) is the F_ST_ values within 50 kb windows separated by 5 kb steps. (DOCX 300 kb)
Additional file 6: Table S5. Names and descriptions of major candidate meat quality related genes. All gene names and descriptions are based on RefSeq. (DOCX 24 kb)

## Abbreviations

GO BP: Gene Ontology Biological Processes
KEGG: Kyoto Encyclopedia of Genes and Genomes
ML: Maximum likelihood
NJ: Neighbor-joining
Ts/Tv: Transition/Transversion ratio
XP-CLR: Cross population composite likelihood ratio
XP-EHH: Cross population extended haplotype homozygosity

## References

[1] Mwai O, Hanotte O, Kwon Y-J, Cho S (2015). African indigenous cattle: unique genetic resources in a rapidly changing world. Asian-Aust J Anim Sci.

[2] Rege J (1999). The state of African cattle genetic resources I. Classification framework and identification of threatened and extinct breeds. Anim Genet Res Inf.

[3] Hanotte O, Dessie T, Kemp S (2010). Time to tap Africa's livestock genomes. Science.

[4] Hansen P (2004). Physiological and cellular adaptations of zebu cattle to thermal stress. Anim Reprod Sci.

[5] Piper EK, Jonsson NN, Gondro C, Lew-Tabor AE, Moolhuijzen P, Vance ME (2009). Immunological profiles of Bos taurus and Bos indicus cattle infested with the cattle tick, Rhipicephalus (Boophilus) microplus. Clin Vaccine Immunol.

[6] Strydom P, Naude R, Smith M, Scholtz M, Van Wyk J (2000). Characterization of indigenous African cattle breeds in relation to carcass characteristics. Anim Sci.

[7] Strydom P (2008). Do indigenous Southern African cattle breeds have the right genetics for commercial production of quality meat?. Meat Sci.

[8] Rege J, Tawah C (1999). The state of African cattle genetic resources II. Geographical distribution, characteristics and uses of present-day breeds and strains. Anim Genet Res Inf.

[9] Grigson C (1991). An African origin for African cattle?—some archaeological evidence. Afr Archaeol Rev.

[10] Strydom P, Frylinck L, Van der Westhuizen J, Burrow H (2008). Growth performance, feed efficiency and carcass and meat quality of tropically adapted breed types from different farming systems in South Africa. Anim Prod Sci.

[11] Strydom P, Frylinck L, Smith M (2011). Variation in meat quality characteristics between Sanga (Bos taurus africanus) and Sanga-derived cattle breeds and between Sanga and Brahman (Bos indicus). Animal.

[12] Gazzola C, O'Neill C, Frisch J (1999). Comparative evaluation of the meat quality of beef cattle breeds of Indian, African and European origins. Anim Sci.

[13] Kamatara K, Mpairwe D, Christensen M, Mutetikka D, Madsen J (2013). Sensory characteristics and tenderness of meat from Ankole bulls: Influence of crossbreeding and feeding system. S Afr J Anim Sci.

[14] Maltin C, Balcerzak D, Tilley R, Delday M (2003). Determinants of meat quality: tenderness. Proc Nutr Soc.

[15] Bernard C, Cassar-Malek I, Le Cunff M, Dubroeucq H, Renand G, Hocquette J-F (2007). New indicators of beef sensory quality revealed by expression of specific genes. J Agric Food Chem.

[16] Mullen A, Stapleton P, Corcoran D, Hamill R, White A (2006). Understanding meat quality through the application of genomic and proteomic approaches. Meat Sci.

[17] Rios Utrera A, Van Vleck LD (2004). Heritability estimates for carcass traits of cattle: a review. Genet Mol Res.

[18] Johnston D, Reverter A, Ferguson D, Thompson J, Burrow H (2003). Genetic and phenotypic characterisation of animal, carcass, and meat quality traits from temperate and tropically adapted beef breeds. 3. Meat quality traits. Aust J Agric Res.

[19] Nielsen R (2005). Molecular signatures of natural selection. Annu Rev Genet.

[20] Bahbahani H, Clifford H, Wragg D, Mbole-Kariuki MN, Van Tassell C, Sonstegard T, et al. Signatures of positive selection in East African Shorthorn Zebu: A genome-wide single nucleotide polymorphism analysis. Sci Rep. 2015;5. 10.1038/srep11729 PMC4486961 26130263

[21] Flori L, Thevenon S, Dayo GK, Senou M, Sylla S, Berthier D (2014). Adaptive admixture in the West African bovine hybrid zone: insight from the Borgou population. Mol Ecol.

[22] Lee H-J, Kim J, Lee T, Son JK, Yoon H-B, Baek K-S (2014). Deciphering the genetic blueprint behind Holstein milk proteins and production. Genome Biol Evol.

[23] Moon S, Kim T-H, Lee K-T, Kwak W, Lee T, Lee S-W (2015). A genome-wide scan for signatures of directional selection in domesticated pigs. BMC Genomics.

[24] Sabeti PC, Varilly P, Fry B, Lohmueller J, Hostetter E, Cotsapas C (2007). Genome-wide detection and characterization of positive selection in human populations. Nature.

[25] Chen H, Patterson N, Reich D (2010). Population differentiation as a test for selective sweeps. Genome Res.

[26] Huang DW, Sherman BT, Lempicki RA (2009). Bioinformatics enrichment tools: paths toward the comprehensive functional analysis of large gene lists. Nucleic Acids Res.

[27] Bindea G, Mlecnik B, Hackl H, Charoentong P, Tosolini M, Kirilovsky A (2009). ClueGO: a Cytoscape plug-in to decipher functionally grouped gene ontology and pathway annotation networks. Bioinformatics.

[28] Korneliussen TS, Moltke I, Albrechtsen A, Nielsen R (2013). Calculation of Tajima’s D and other neutrality test statistics from low depth next-generation sequencing data. BMC Bioinformatics.

[29] Oleksyk TK, Smith MW, O'Brien SJ (2010). Genome-wide scans for footprints of natural selection. Phil Trans R Soc B.

[30] Qanbari S, Simianer H (2014). Mapping signatures of positive selection in the genome of livestock. Livest Sci.

[31] Koohmaraie M, Kent MP, Shackelford SD, Veiseth E, Wheeler TL (2002). Meat tenderness and muscle growth: is there any relationship?. Meat Sci.

[32] Guillemin N, Bonnet M, Jurie C, Picard B (2011). Functional analysis of beef tenderness. J Proteomics.

[33] Schoeman S (1989). Recent research into the production potential of indigenous cattle with special reference to the Sanga. S Afr J Anim Sci.

[34] Gao Y, Zhang Y, Jiang H, Xiao S, Wang S, Ma Q (2011). Detection of differentially expressed genes in the longissimus dorsi of Northeastern indigenous and large white pigs. Genet Mol Res.

[35] Damon M, Wyszynska-Koko J, Vincent A, Herault F, Lebret B (2012). Comparison of muscle transcriptome between pigs with divergent meat quality phenotypes identifies genes related to muscle metabolism and structure. PLoS One.

[36] Hamill RM, McBryan J, McGee C, Mullen AM, Sweeney T, Talbot A (2012). Functional analysis of muscle gene expression profiles associated with tenderness and intramuscular fat content in pork. Meat Sci.

[37] Jiang C, Shi P, Li S, Dong R, Tian J, Wei J (2010). Gene expression profiling of skeletal muscle of nursing piglets. Int J Biol Sci.

[38] Ponsuksili S, Murani E, Phatsara C, Schwerin M, Schellander K, Wimmers K (2009). Porcine muscle sensory attributes associate with major changes in gene networks involving CAPZB, ANKRD1, and CTBP2. Funct Integr Genomics.

[39] Chang K (2007). Key signalling factors and pathways in the molecular determination of skeletal muscle phenotype. Animal.

[40] Rolf M, Taylor J, Schnabel R, McKay S, McClure M, Northcutt S (2012). Genome‐wide association analysis for feed efficiency in Angus cattle. Anim Genet.

[41] Joo S, Kim G, Hwang Y, Ryu Y (2013). Control of fresh meat quality through manipulation of muscle fiber characteristics. Meat Sci.

[42] Culler R, Smith G, Cross H (1978). Relationship of myofibril fragmentation index to certain chemical, physical and sensory characteristics of bovine longissimus muscle. J Food Sci.

[43] Xu Y, Qian H, Feng X, Xiong Y, Lei M, Ren Z (2012). Differential proteome and transcriptome analysis of porcine skeletal muscle during development. J Proteomics.

[44] Pyle WG, Hart MC, Cooper JA, Sumandea MP, de Tombe PP, Solaro RJ (2002). Actin capping protein an essential element in protein kinase signaling to the myofilaments. Circ Res.

[45] Schellander K (2010). Identifying genes associated with quantitative traits in pigs: integrating quantitative and molecular approaches for meat quality. Ital J Anim Sci.

[46] Ghosh M, Sodhi S, Song KD, Kim J, Mongre R, Sharma N (2015). Evaluation of body growth and immunity‐related differentially expressed genes through deep RNA sequencing in the piglets of Jeju native pig and Berkshire. Anim Genet.

[47] Cho I, Park H, Yoo C, Lee G, Lim H, Lee J (2011). QTL analysis of white blood cell, platelet and red blood cell‐related traits in an F2 intercross between Landrace and Korean native pigs. Anim Genet.

[48] Lu Y, Chen S, Liu W, Hou Z, Xu G, Yang N (2012). Polymorphisms in Wnt signaling pathway genes are significantly associated with chicken carcass traits. Poult Sci.

[49] Wu H, Zhao S, Fan B (2010). Investigation of effects of the MKK3 and MKK6 genes on meat production traits in the pig (Brief Report). Archiv Tierzucht.

[50] Yin H, Gui Y, Du G, Frohman MA, Zheng X-L (2010). Dependence of phospholipase D1 multi-monoubiquitination on its enzymatic activity and palmitoylation. J Biol Chem.

[51] Clark KA, McElhinny AS, Beckerle MC, Gregorio CC (2002). Striated muscle cytoarchitecture: an intricate web of form and function. Annu Rev Cell Dev Biol.

[52] Teltathum T, Mekchay S. Relationships between Pectoralis muscle proteomes and shear force in Thai indigenous chicken meat. Kasetsart J (Nat Sci). 2010.

[53] Wimmers K, Murani E, Ngu N, Schellander K, Ponsuksili S (2007). Structural and functional genomics to elucidate the genetic background of microstructural and biophysical muscle properties in the pig. J Anim Breed Genet.

[54] Lonergan EH, Zhang W, Lonergan SM (2010). Biochemistry of postmortem muscle—Lessons on mechanisms of meat tenderization. Meat Sci.

[55] da Costa N, Edgar J, Ooi P-T, Su Y, Meissner JD, Chang K-C (2007). Calcineurin differentially regulates fast myosin heavy chain genes in oxidative muscle fibre type conversion. Cell Tissue Res.

[56] Nakamura Y, Kanemarum K, Fukami K (2013). Physiological functions of phospholipase Cδ1 and phospholipase Cδ3. Adv Biol Regul.

[57] Duncan RE, Sarkadi-Nagy E, Jaworski K, Ahmadian M, Sul HS (2008). Identification and functional characterization of adipose-specific phospholipase A2 (AdPLA). J Biol Chem.

[58] Wang W, Xue W, Jin B, Zhang X, Ma F, Xu X (2013). Candidate gene expression affects intramuscular fat content and fatty acid composition in pigs. J Appl Genet.

[59] Chan EK, Reverter A (2007). Integrating whole-genome genetic-association studies with gene expression data to prioritise candidate genes affecting intramuscular fat in beef cattle traits. Proc Assoc Advmt Anim Breed Genet.

[60] Sung MK, Bae YJ (2010). Linking obesity to colorectal cancer: application of nutrigenomics. Biotechnol J.

[61] Cánovas A, Quintanilla R, Amills M, Pena RN (2010). Muscle transcriptomic profiles in pigs with divergent phenotypes for fatness traits. BMC Genomics.

[62] Roux P-F, Boitard S, Blum Y, Parks B, Montagner A, Mouisel E (2015). Combined QTL and selective swe1ep mappings with coding SNP annotation and cis-eQTL analysis revealed PARK2 and JAG2 as new candidate genes for adiposity regulation. G3 (Bethesda).

[63] Puri V, Virbasius J, Guilherme A, Czech M (2008). RNAi screens reveal novel metabolic regulators: RIP140, MAP4k4 and the lipid droplet associated fat specific protein (FSP) 27. Acta Physiol (Oxf).

[64] Cho E-S, Jeon H-J, Lee S-W, Park J-W, Raveendar S, Jang G-W (2013). Association of a Pyruvate Kinase M2 (PKM2) Polymorphism with Back Fat Thickness in Berkshire Pigs. J Anim Sci Technol.

[65] Corella D, Ordovas JM (2005). Single nucleotide polymorphisms that influence lipid metabolism: interaction with dietary factors. Annu Rev Nutr.

[66] Perez R, Cañón J, Dunner S (2010). Genes associated with long-chain omega-3 fatty acids in bovine skeletal muscle. J Appl Genet.

[67] Mancini R, Hunt M (2005). Current research in meat color. Meat Sci.

[68] Khan AA, Quigley JG (2013). Heme and FLVCR-related transporter families SLC48 and SLC49. Mol Aspects Med.

[69] Borchers N, Otto G, Kalm E (2007). Genetic relationship of drip loss to further meat quality traits in purebred Pietrains. Arch Tierz.

[70] Ponsuksili S, Jonas E, Murani E, Phatsara C, Srikanchai T, Walz C (2008). Trait correlated expression combined with expression QTL analysis reveals biological pathways and candidate genes affecting water holding capacity of muscle. BMC Genomics.

[71] Shen L, Lei H, Zhang S, Li X, Li M, Jiang X (2014). The comparison of energy metabolism and meat quality among three pig breeds. Anim Sci J.

[72] Sherman E, Nkrumah J, Moore S (2010). Whole genome single nucleotide polymorphism associations with feed intake and feed efficiency in beef cattle. J Anim Sci.

[73] Chen Y, Gondro C, Quinn K, Herd R, Parnell P, Vanselow B (2011). Global gene expression profiling reveals genes expressed differentially in cattle with high and low residual feed intake. Anim Genet.

[74] Fontanesi L, Davoli R, Costa LN, Beretti F, Scotti E, Tazzoli M (2008). Investigation of candidate genes for glycolytic potential of porcine skeletal muscle: Association with meat quality and production traits in Italian Large White pigs. Meat Sci.

[75] Do DN, Ostersen T, Strathe AB, Mark T, Jensen J, Kadarmideen HN (2014). Genome-wide association and systems genetic analyses of residual feed intake, daily feed consumption, backfat and weight gain in pigs. BMC Genet.

[76] Santana M, Kadarmideen H, Pant S, Alexandre P, Junior GO, Gomes R (2014). Systems genetics investigations for feed intake, feed efficiency and performance in Nellore (Bos indicus) Cattle. 10th World Congress on Genetics Applied to Livestock Production, August 17–22, 2014.

[77] Do DN, Strathe AB, Ostersen T, Pant SD, Kadarmideen HN (2014). Genome-wide association and pathway analysis of feed efficiency in pigs reveal candidate genes and pathways for residual feed intake. Front Genet.

[78] Choquette AC, Bouchard L, Drapeau V, Lemieux S, Tremblay A, Bouchard C (2012). Association between olfactory receptor genes, eating behavior traits and adiposity: results from the Quebec family study. Physiol Behav.

[79] Zhou N, Lee WR, Abasht B (2015). Messenger RNA sequencing and pathway analysis provide novel insights into the biological basis of chickens’ feed efficiency. BMC Genomics.

[80] Ndumu D, Baumung R, Hanotte O, Wurzinger M, Okeyo M, Jianlin H (2008). Genetic and morphological characterisation of the Ankole Longhorn cattle in the African Great Lakes region. Genet Sel Evol.

[81] Kugonza D, Nabasirye M, Mpairwe D, Hanotte O, Okeyo A (2011). Productivity and morphology of Ankole cattle in three livestock production systems in Uganda. Anim Genet Resour.

[82] Rewe T, Herold P, Kahi A, Valle Zárate A (2009). Breeding indigenous cattle genetic resources for beef production in Sub-Saharan Africa. Outlook Agric.

[83] Andrews S. FastQC: a quality control tool for high throughput sequence data. Reference Source. 2010.

[84] Langmead B, Salzberg SL (2012). Fast gapped-read alignment with Bowtie 2. Nat Methods.

[85] Li H, Handsaker B, Wysoker A, Fennell T, Ruan J, Homer N (2009). The sequence alignment/map format and SAMtools. Bioinformatics.

[86] McKenna A, Hanna M, Banks E, Sivachenko A, Cibulskis K, Kernytsky A (2010). The Genome Analysis Toolkit: a MapReduce framework for analyzing next-generation DNA sequencing data. Genome Res.

[87] Browning SR, Browning BL (2007). Rapid and accurate haplotype phasing and missing-data inference for whole-genome association studies by use of localized haplotype clustering. Am J Hum Genet.

[88] Felsenstein J (1981). Evolutionary trees from DNA sequences: a maximum likelihood approach. J Mol Evol.

[89] Schmidt HA, Strimmer K, Vingron M, von Haeseler A (2002). TREE-PUZZLE: maximum likelihood phylogenetic analysis using quartets and parallel computing. Bioinformatics.

[90] Strimmer K, Von Haeseler A (1996). Quartet puzzling: a quartet maximum-likelihood method for reconstructing tree topologies. Mol Biol Evol.

[91] Saitou N, Nei M (1987). The neighbor-joining method: a new method for reconstructing phylogenetic trees. Mol Biol Evol.

[92] Felsenstein J (1993). PHYLIP (phylogeny inference package), 3.5 c ed.

[93] Kimura M (1980). A simple method for estimating evolutionary rate of base substitution through comparative studies of nucleotide sequence. Mol Evol.

[94] Felsenstein J (1985). Confidence limits on phylogenies: an approach using the bootstrap. Evolution.

[95] Pickrell JK, Coop G, Novembre J, Kudaravalli S, Li JZ, Absher D (2009). Signals of recent positive selection in a worldwide sample of human populations. Genome Res.

[96] Danecek P, Auton A, Abecasis G, Albers CA, Banks E, DePristo MA (2011). The variant call format and VCFtools. Bioinformatics.

