# Supplementary material for: Whole genome scan reveals the genetic signature of African Ankole cattle breed and potential for higher quality beef
Source: BMC Genet. 2017 Feb 9;18:11. doi: 10.1186/s12863-016-0467-1 (PMC5301378; doi:10.1186/s12863-016-0467-1)
Supplement: Additional file 5: Figure S1. — Tajima’s D and FST plot of positively selected gene regions in Sanga and indicus cattle populations. The Tajima’s D plot for each gene region (upper plot for each gene) is the Tajima’s D value within 50 kb window plotted for both populations. The smaller (negative) Tajima’s D value in Sanga population shows that the gene region considered is under positive selection. The FST plot (lower plot for each gene) is the FST values within 50 kb windows separated by 5 kb steps. (DOCX 300 kb) [file 12863_2016_467_MOESM5_ESM.docx]

| 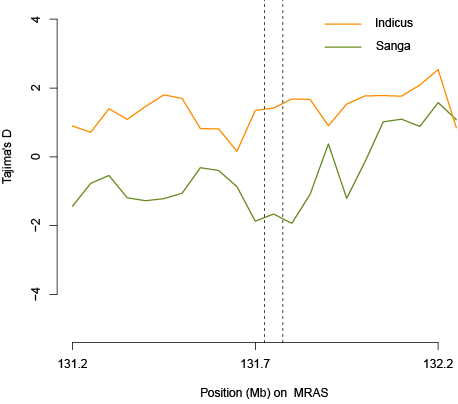  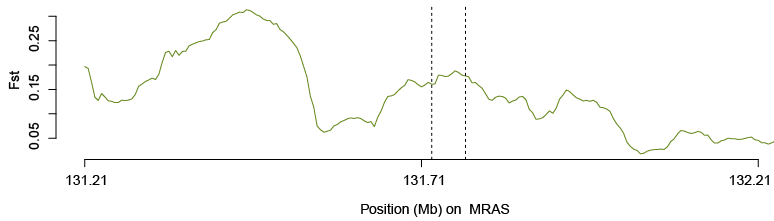 |
| --- |
| 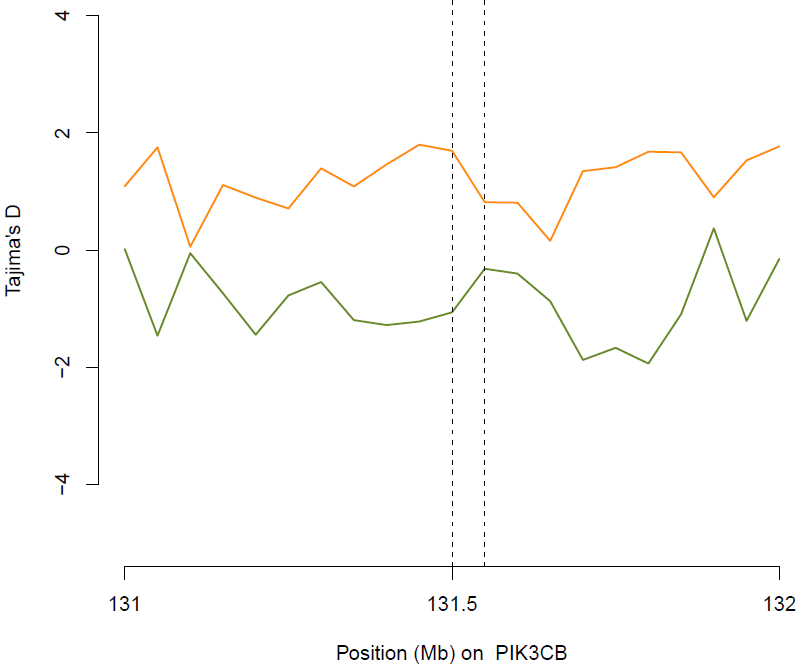  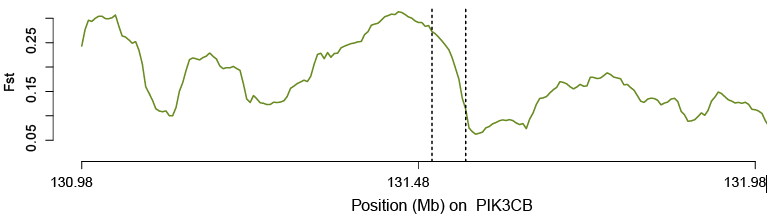 |
| 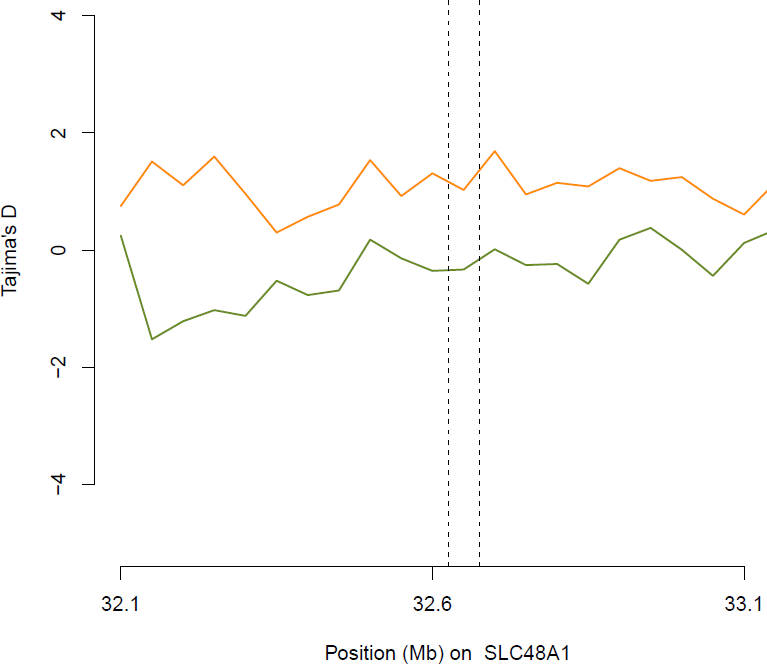  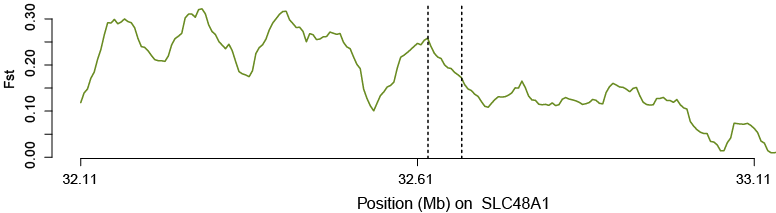 |
| 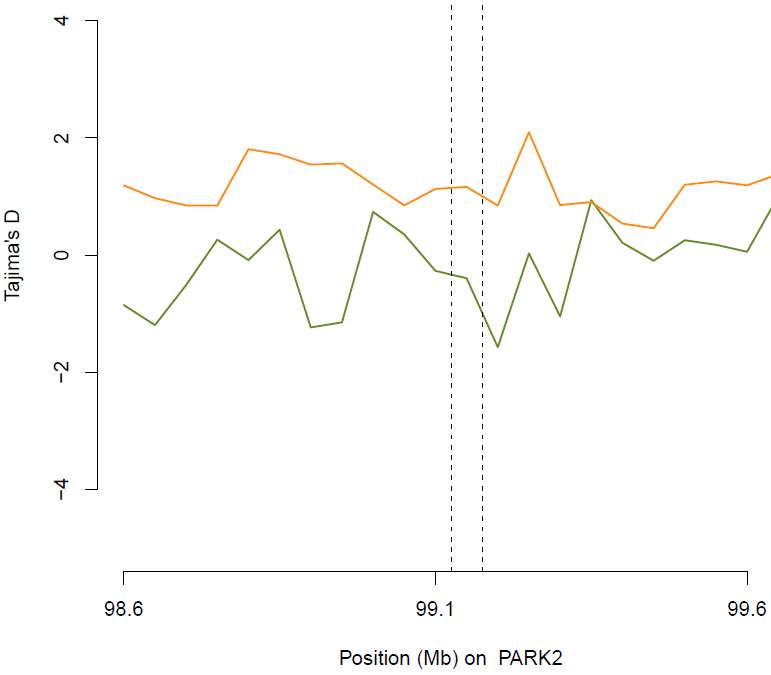  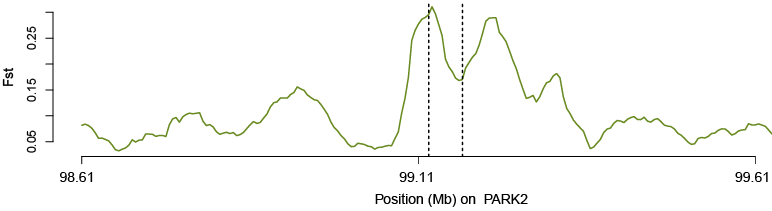 |
| 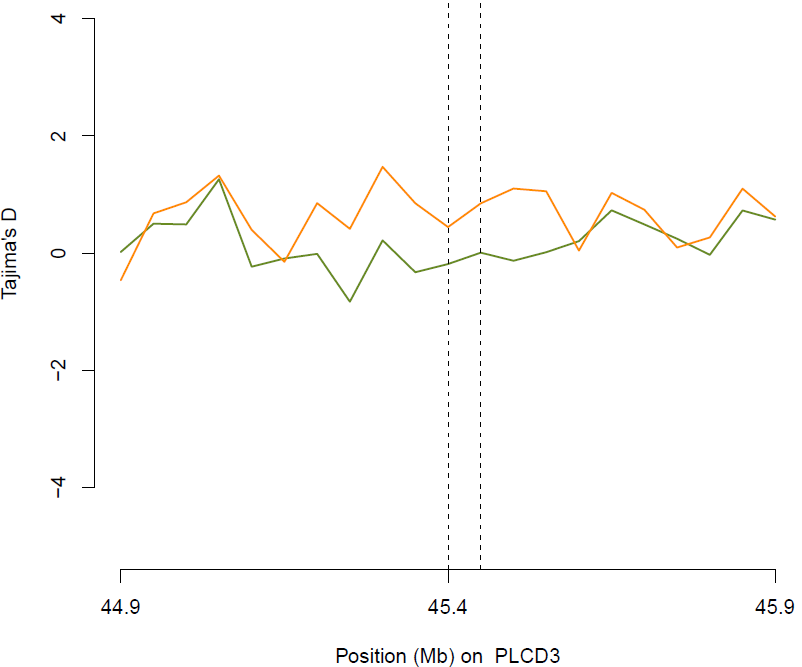  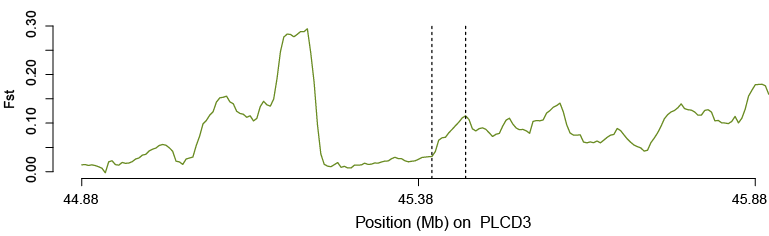 |
| 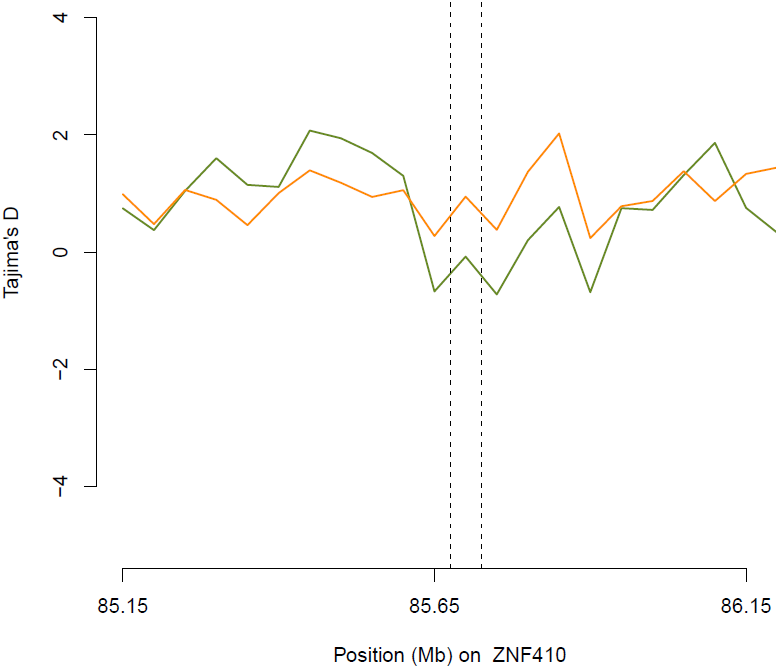  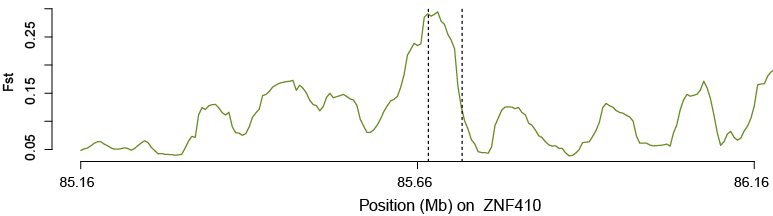 |

**Additional file 5: Figure S1.** Fst and Tajima’s D plot of positively selected gene regions in Ankole and Indicus cattle populations. The Tajima’s D plot for each gene region (upper plot for each gene) is the Tajima’s D value with in 50kb window plotted for both populations. The smaller (negative) Tajima’s D value in Ankole population shows that the gene region considered is positively selected. The Fst plot (under for each gene) is the Fst values within 50 kb windows separated by 5000 steps.
