# Supplementary material for: Whole genome scan reveals the genetic signature of African Ankole cattle breed and potential for higher quality beef
Source: BMC Genet. 2017 Feb 9;18:11. doi: 10.1186/s12863-016-0467-1 (PMC5301378; doi:10.1186/s12863-016-0467-1)
Supplement: Additional file 6: Table S5. — Names and descriptions of major candidate meat quality related genes. All gene names and descriptions are based on RefSeq. (DOCX 24 kb) [file 12863_2016_467_MOESM6_ESM.docx]

Additional file 6: Table S6. Descriptions of major candidate genes. All gene descriptions are based on RefSeq (Pruitt et al., 2007) and GeneCards (Safran et al., 2010)

| Candidate genes | Description | Function | Related Pathway |
| --- | --- | --- | --- |
| CAPZB | Capping Protein (Actin Filament) Muscle Z-Line, Beta | The capping protein is a heterodimeric actin capping protein that blocks actin filament assembly and disassembly at the fast growing (barbed) filament ends and functions in regulating actin filament dynamics as well as in stabilizing actin filament lengths in muscle and nonmuscle cells. | [Hemostasis](http://pathcards.genecards.org/card/hemostasis) and [Factors involved in megakaryocyte development and platelet production](http://pathcards.genecards.org/card/factors_involved_in_megakaryocyte_development_and_platelet_production) |
| PLA2G2A | Phospholipase A2, Group IIA (Platelets, Synovial Fluid) | Thought to participate in the regulation of the phospholipid metabolism in biomembranes including eicosanoid biosynthesis. Catalyzes the calcium-dependent hydrolysis of the 2-acyl groups in 3-sn-phosphoglycerides | [Ras signaling pathway](http://pathcards.genecards.org/card/ras_signaling_pathway) and [PAK Pathway](http://pathcards.genecards.org/card/pak_pathway). |
| COL9A2 | Collagen, Type IX, Alpha 2 | Structural component of hyaline cartilage and vitreous of the eye | [Signaling by FGFR](http://pathcards.genecards.org/card/signaling_by_fgfr) and [Signaling by GPCR](http://pathcards.genecards.org/card/signaling_by_gpcr). |
| LIMA | LIM Domain And Actin Binding 1 OR  Epithelial Protein Lost In Neoplasm Beta | Binds to actin monomers and filaments. Increases the number and size of actin stress fibers and inhibits membrane ruffling. Inhibits actin filament depolymerization. Bundles actin filaments, delays filament nucleation and reduces formation of branched filaments. | [Stabilization and expansion of the E-cadherin adherens junction](http://pathcards.genecards.org/card/stabilization_and_expansion_of_the_e-cadherin_adherens_junction) |
| MB | Myoglobin | Serves as a reserve supply of oxygen and facilitates the movement of oxygen within muscles | [Effects of nitric oxide](http://pathcards.genecards.org/card/effects_of_nitric_oxide) |
| APOL6 | Apolipoprotein L6 | May affect the movement of lipids in the cytoplasm or allow the binding of lipids to organelles | [Cholesterol and Sphingolipids transport / Recycling to plasma membrane in lung (normal and CF)](http://pathcards.genecards.org/card/cholesterol_and_sphingolipids_transport__recycling_to_plasma_membrane_in_lung_(normal_and_cf)). |
| PDGFRA | Platelet-Derived Growth Factor Receptor, Alpha Polypeptide | Platelet-derived growth factor receptors (PDGFRs) are catalytic receptors that have intracellular tyrosine kinase activity. They have roles in the regulation of many biological processes including embryonic development, angiogenesis, cell proliferation and differentiation, and contribute to the pathophysiology of some diseases, including cancer.  This activates the intracellular kinase activity, initiating intracellular signaling through the MAPK, PI 3-K and PKCgamma pathways. | [PI3K-Akt signaling pathway](http://pathcards.genecards.org/card/pi3k-akt_signaling_pathway) and [PI-3K cascade](http://pathcards.genecards.org/card/pi-3k_cascade) |
| PARK2 | Parkin RBR E3 Ubiquitin Protein Ligase | Mediates monoubiquitination and polyubiquitination.  Limits the production of reactive oxygen species (ROS). | [Class I MHC mediated antigen processing and presentation](http://pathcards.genecards.org/card/class_i_mhc_mediated_antigen_processing_and_presentation) and [Alpha-synuclein signaling](http://pathcards.genecards.org/card/alpha-synuclein_signaling). |
| MAP3K5 | Mitogen-Activated Protein Kinase Kinase Kinase 5 | Serine/threonine kinase which acts as an essential component of the MAP kinase signal transduction pathway. | [MAPK signaling pathway](http://pathcards.genecards.org/card/mapk_signaling_pathway) and [Development FGFR signaling pathway](http://pathcards.genecards.org/card/development_fgfr_signaling_pathway) |
| ZNF410 | Zinc Finger Protein 410 | Transcription factor that activates transcription of matrix-remodeling genes such as MMP1 during fibroblast senescence. |  |
| AHSA1 | Activator Of 90 KDa Heat Shock Protein ATPase Homolog 1 | Cochaperone that stimulates HSP90 ATPase activity (By similarity) | [Mechanisms of CFTR activation by S-nitrosoglutathione (normal and CF)](http://pathcards.genecards.org/card/mechanisms_of_cftr_activation_by_s-nitrosoglutathione_(normal_and_cf)) |
| PKM2 | Pyruvate Kinase, Muscle | Glycolytic enzyme that catalyzes the transfer of a phosphoryl group from phosphoenolpyruvate (PEP) to ADP, generating ATP. | [Metabolism](http://pathcards.genecards.org/card/metabolism) and [Carbon metabolism](http://pathcards.genecards.org/card/carbon_metabolism). |
| MAP4K4 | Mitogen-Activated Protein Kinase Kinase Kinase Kinase 4 | Serine/threonine kinase that may play a role in the response to environmental stress and cytokines such as TNF-alpha. | [MAPK signaling pathway](http://pathcards.genecards.org/card/mapk_signaling_pathway) and [TGF-Beta Pathway](http://pathcards.genecards.org/card/tgf-beta_pathway) |
| WWP1 | WW Domain Containing E3 Ubiquitin Protein Ligase 1 | Ubiquitinates and promotes degradation | [PI-3K cascade](http://pathcards.genecards.org/card/pi-3k_cascade) and [Signaling by GPCR](http://pathcards.genecards.org/card/signaling_by_gpcr) |
| MAPKAPK2 | Mitogen-Activated Protein Kinase-Activated Protein Kinase 2 | Stress-activated serine/threonine-protein kinase involved in cytokines production, endocytosis, reorganization of the cytoskeleton, cell migration, cell cycle control, chromatin remodeling, DNA damage response and transcriptional regulation. | [Signaling by FGFR](http://pathcards.genecards.org/card/signaling_by_fgfr) and [Signaling by FGFR](http://pathcards.genecards.org/card/signaling_by_fgfr). |
| MAP2K3 | Mitogen-Activated Protein Kinase Kinase 3 | Catalyzes the concomitant phosphorylation of a threonine and a tyrosine residue in the MAP kinase p38. | [MAPK signaling pathway](http://pathcards.genecards.org/card/mapk_signaling_pathway) and [GPCR Pathway](http://pathcards.genecards.org/card/gpcr_pathway). |
| PLCD3 | Phospholipase C, Delta 3 | Phospholipases are a group of enzymes that hydrolyze phospholipids into fatty acids and other lipophilic molecules. Phospholipases are ubiquitously expressed and have diverse biological functions including roles in inflammation, cell growth, signaling and death and maintenance of membrane phospholipids. | [GPCR Pathway](http://pathcards.genecards.org/card/gpcr_pathway) and [Akt Signaling](http://pathcards.genecards.org/card/akt_signaling) |
| PLCD1 | Phospholipase C, Delta 1 | Phospholipases are a group of enzymes that hydrolyze phospholipids into fatty acids and other lipophilic molecules. Phospholipases are ubiquitously expressed and have diverse biological functions including roles in inflammation, cell growth, signaling and death and maintenance of membrane phospholipids. | [GPCR Pathway](http://pathcards.genecards.org/card/gpcr_pathway) and [Akt Signaling](http://pathcards.genecards.org/card/akt_signaling) |
| ROCK1 | Rho-Associated, Coiled-Coil Containing Protein Kinase 1 | Rho-kinases are serine/threonine kinases that are activated by RhoA GTPases, and are key modulators of processes involving cytoskeletal rearrangement such focal adhesion formation, cell motility and tumor cell invasion. ROCKs phosphorylate a number of proteins involved in actin filament assembly and contraction | [RhoGDI Pathway](http://pathcards.genecards.org/card/rhogdi_pathway) and [Signaling by GPCR](http://pathcards.genecards.org/card/signaling_by_gpcr). |
| PRKG1 | Protein Kinase, CGMP-Dependent, Type I | Serine/threonine protein kinase that acts as key mediator of the nitric oxide (NO)/cGMP signaling pathway. Activation of PRKG1 by NO signaling alters also gene expression in a number of tissues. In smooth muscle cells, increased cGMP and PRKG1 activity influence expression of smooth muscle-specific contractile proteins, levels of proteins in the NO/cGMP signaling pathway, down-regulation of the matrix proteins osteopontin and thrombospondin-1 to limit smooth muscle cell migration and phenotype | [Signaling by GPCR](http://pathcards.genecards.org/card/signaling_by_gpcr) and [Class I MHC mediated antigen processing and presentation](http://pathcards.genecards.org/card/class_i_mhc_mediated_antigen_processing_and_presentation). |
| PIK3CB | Phosphatidylinositol-4,5-Bisphosphate 3-Kinase, Catalytic Subunit Beta | PI 3-Kinases are family of lipid kinases capable of phosphorylating the 3'OH of the inositol ring of phosphoinositides. They are responsible for coordinating a diverse range of cell functions including proliferation, cell survival, degranulation, vesicular trafficking and cell migration. | [PI3K-Akt signaling pathway](http://pathcards.genecards.org/card/pi3k-akt_signaling_pathway) and [PI-3K cascade](http://pathcards.genecards.org/card/pi-3k_cascade). |
| NFATC2 | Nuclear Factor Of Activated T-Cells, Cytoplasmic, Calcineurin-Dependent 2 | Plays a role in the inducible expression of cytokine genes in T-cells, especially in the induction of the IL-2, IL-3, IL-4, TNF-alpha or GM-CSF. Promotes invasive migration through the activation of GPC6 expression and WNT5A signaling pathway. | [PI-3K cascade](http://pathcards.genecards.org/card/pi-3k_cascade) and [GPCR Pathway](http://pathcards.genecards.org/card/gpcr_pathway) |
| ATP8A1 | ATPase, Aminophospholipid Transporter (APLT), Class I, Type 8A, Member 1 | Play a role in regulation of cell migration probably involving flippase-mediated translocation of phosphatidylethanolamine (PE) at the plasma membrane. | [Ion channel transport](http://pathcards.genecards.org/card/ion_channel_transport) and [Transport of glucose and other sugars, bile salts and organic acids, metal ions and amine compounds](http://pathcards.genecards.org/card/transport_of_glucose_and_other_sugars_bile_salts_and_organic_acids_metal_ions_and_amine_compounds) |
| MRAS | Muscle RAS Oncogene Homolog | May serve as an important signal transducer for a novel upstream stimuli in controlling cell proliferation. Weakly activates the MAP kinase pathway. | [MAPK signaling pathway](http://pathcards.genecards.org/card/mapk_signaling_pathway) and [GPCR Pathway](http://pathcards.genecards.org/card/gpcr_pathway). |
| OR2D2 | Olfactory Receptor, Family 2, Subfamily D, Member 2 | Odorant receptor (Potential). May be involved in taste perception. | [Signaling by GPCR](http://pathcards.genecards.org/card/signaling_by_gpcr) and [Signaling by GPCR](http://pathcards.genecards.org/card/signaling_by_gpcr) |
| OR10A4 | Olfactory Receptor, Family 10, Subfamily A, Member 4 | Odorant receptor (Potential). May be involved in taste perception. | [Signaling by GPCR](http://pathcards.genecards.org/card/signaling_by_gpcr) and [Signaling by GPCR](http://pathcards.genecards.org/card/signaling_by_gpcr) |
| OR2D3 | Olfactory Receptor, Family 2, Subfamily D, Member 3 | Odorant receptor (Potential). May be involved in taste perception. | [Signaling by GPCR](http://pathcards.genecards.org/card/signaling_by_gpcr) and [Signaling by GPCR](http://pathcards.genecards.org/card/signaling_by_gpcr) |
